# Assessment of Clinical Metadata on the Accuracy of Retinal Fundus Image Labels in Diabetic Retinopathy in Uganda: Case-Crossover Study Using the Multimodal Database of Retinal Images in Africa

**DOI:** 10.2196/59914

**Published:** 2024-09-18

**Authors:** Simon Arunga, Katharine Elise Morley, Teddy Kwaga, Michael Gerard Morley, Luis Filipe Nakayama, Rogers Mwavu, Fred Kaggwa, Julius Ssempiira, Leo Anthony Celi, Jessica E Haberer, Celestino Obua

**Affiliations:** 1 Department of Ophthalmology Mbarara University of Science and Technology Mbarara Uganda; 2 Massachusetts General Hospital Center for Global Health Department of Medicine Harvard Medical School Boston, MA United States; 3 Harvard Ophthalmology AI Lab Massachusetts Eye and Ear Infirmary Harvard Medical School Boston, MA United States; 4 Ophthalmology Department Sao Paulo Federal University Sao Paulo Brazil; 5 Laboratory for Computational Physiology Massachusetts Institute of Technology Cambridge, MA United States; 6 Faculty of Computing and Informatics Department of Information Technology Mbarara University of Science and Technology Mbarara Uganda; 7 Faculty of Computing and Informatics Department of Computer Science Mbarara University of Science and Technology Mbarara Uganda; 8 School of Public Health Makerere University Kampala Uganda; 9 Division of Pulmonary, Critical Care and Sleep Medicine Beth Israel Deaconess Medical Center Harvard Medical School Boston, MA United States; 10 Department of Biostatistics Harvard T.H. Chan School of Public Health Boston, MA United States; 11 Department of Pharmacology Mbarara University of Science and Technology Mbarara Uganda

**Keywords:** image labeling, metadata, diabetic retinopathy, assessment, bias, multimodal database, retinal images, Africa, African, artificial intelligence, AI, screening algorithms, screening, algorithms, diabetic, diabetes, treatment, sensitivity, clinical images

## Abstract

**Background:**

Labeling color fundus photos (CFP) is an important step in the development of artificial intelligence screening algorithms for the detection of diabetic retinopathy (DR). Most studies use the International Classification of Diabetic Retinopathy (ICDR) to assign labels to CFP, plus the presence or absence of macular edema (ME). Images can be grouped as referrable or nonreferrable according to these classifications. There is little guidance in the literature about how to collect and use metadata as a part of the CFP labeling process.

**Objective:**

This study aimed to improve the quality of the Multimodal Database of Retinal Images in Africa (MoDRIA) by determining whether the availability of metadata during the image labeling process influences the accuracy, sensitivity, and specificity of image labels. MoDRIA was developed as one of the inaugural research projects of the Mbarara University Data Science Research Hub, part of the Data Science for Health Discovery and Innovation in Africa (DS-I Africa) initiative.

**Methods:**

This is a crossover assessment with 2 groups and 2 phases. Each group had 10 randomly assigned labelers who provided an ICDR score and the presence or absence of ME for each of the 50 CFP in a test image with and without metadata including blood pressure, visual acuity, glucose, and medical history. Specificity and sensitivity of referable retinopathy were based on ICDR scores, and ME was calculated using a 2-sided *t* test. Comparison of sensitivity and specificity for ICDR scores and ME with and without metadata for each participant was calculated using the Wilcoxon signed rank test. Statistical significance was set at *P*<.05.

**Results:**

The sensitivity for identifying referrable DR with metadata was 92.8% (95% CI 87.6-98.0) compared with 93.3% (95% CI 87.6-98.9) without metadata, and the specificity was 84.9% (95% CI 75.1-94.6) with metadata compared with 88.2% (95% CI 79.5-96.8) without metadata. The sensitivity for identifying the presence of ME was 64.3% (95% CI 57.6-71.0) with metadata, compared with 63.1% (95% CI 53.4-73.0) without metadata, and the specificity was 86.5% (95% CI 81.4-91.5) with metadata compared with 87.7% (95% CI 83.9-91.5) without metadata. The sensitivity and specificity of the ICDR score and the presence or absence of ME were calculated for each labeler with and without metadata. No findings were statistically significant.

**Conclusions:**

The sensitivity and specificity scores for the detection of referrable DR were slightly better without metadata, but the difference was not statistically significant. We cannot make definitive conclusions about the impact of metadata on the sensitivity and specificity of image labels in our study. Given the importance of metadata in clinical situations, we believe that metadata may benefit labeling quality. A more rigorous study to determine the sensitivity and specificity of CFP labels with and without metadata is recommended.

## Introduction

### Background

Imaging examinations in ophthalmology serve as a tool for diagnosing and following up ocular pathologies and play a critical role in the management of diabetic retinopathy (DR). Retinal color fundus photos (CFP)_specifically capture the ocular posterior segment, comprising the retina, optic disc, macula, and vessels, offering crucial information about ocular and systemic health during ophthalmological examinations [1]. Diabetes is a global epidemic, affecting more than 500 million people in 2021 and a projected 783 million by 2045, with DR as the most common complication of systemic diabetes [2]. Retinal CFPs have been used for screening of referable cases, optimizing the referral process worldwide, and more recently they have been used in the development of artificial intelligence (AI) algorithms for automatic DR screening [3].

### Classification of Diabetic Retinopathy

In DR screening algorithms developed using supervised machine learning [4], an important step in the process is labeling the CFPs; these labels indicate the presence and severity of DR and macular edema (ME) for training the AI model. Most studies use a 2-image capturing protocol using the International Classification of Diabetic Retinopathy (ICDR) [5], which has 5 levels of severity (Table 1), that are, 0=no retinopathy, 1=microaneurysms only, 2=hemorrhages, 3=proliferative, and 4=proliferative retinopathy. It has been proven effective in comparison with the gold standard Early Treatment Diabetic Retinopathy Study (ETDRS) field protocol [6]. Individuals with preproliferative (3) and proliferative (4) retinopathy are candidates for treatment intervention with laser, antivascular endothelial growth factors drugs or surgery. The presence of ME is another important criterion for treatment intervention. A key goal for AI screening algorithms is to identify patients with DR who need referral for potential treatment.

**Table 1:** International Classification of Diabetic Retinopathy [5].

Levels			Classifications
**ICDR^a^** **severity level**			
	0	No retinopathy-no abnormalities	
	1	Mild nonproliferative retinopathy-microaneurysm or microaneurysm or microaneurysms only	
	2	Moderate nonproliferative retinopathy-more than just microaneurysm or microaneurysms but less than severe nonproliferative diabetic retinopathy	
	3	Severe nonproliferative or preproliferative retinopathy: any of the following: >20 intraretinal hemorrhages in each of 4 quadrants, venous beading in ≥2 quadrants, intraretinal microvascular abnormalities in ≥1 quadrant, and no signs of proliferative retinopathy	
	4	Proliferative retinopathy-one or more of the following: neovascularization vitreous or preretinal hemorrhages	
**Macula edema**			Exudates or apparent thickening within one disc diameter from the fovea

^a^ICDR: International Classification of Diabetic Retinopathy.

### Background on Fundus Image Labeling and Use of Metadata for the Development of an AI Algorithm

Labeling large numbers of CFPs has many challenges. Strategies including recruiting highly trained retinal specialists, comprehensive ophthalmologists [7], professional labelers, and crowdsourcing using labelers with different backgrounds and experience [8], and more recently, unsupervised learning with deep learning algorithms [9] were used. Another variable is the availability and use of metadata during the labeling process. Metadata for medical imaging can include information generated from the imaging device and process itself such as order codes and image files, along with other biomarkers, demographics, and clinical information related to the image [10]. When an electronic medical record is available, the medical history, diagnostic results, and the clinical assessment and plan may be linked to the image. The actual image interpretation may also be present as in the case of radiology or pathology reports. In the absence of an integrated electronic record, as is typically the case in low-resource settings, any additional clinical information must be collected separately and linked to the image.

The use of local data is crucial for AI development and validation, yet automated systems face a critical risk of biased decisions based on this information [11]. In practice, the clinician makes a diagnosis using all the available information about the patient including history, examination findings, diagnostic tests, and imaging. But labeling is frequently done with only the image (ie, no additional clinical metadata) [12,13]. In their paper on image labeling quality control, Freeman et al [14], reported that the gap between the clinical and labeling contexts is a challenge in optimizing the accuracy of labels. The label tends to be given as an overall impression of the findings. They stressed the importance of having labeling criteria and guidelines explicitly focused on the labeling task to improve consistency and inferred that it does not include other clinical information. Alternatively, Kondylakis et al [10], state that metadata are essential for the correct use and interpretation of medical images and stress the importance of data harmonization to use this information in the development of AI models. The importance of incorporating clinical information as a multimodal data stream has been increasingly recognized in the development of radiology algorithms [15,16]. The availability of correct clinical information has been shown to improve the interpretations of diagnostic tests [17] accuracy of computerized tomography interpretation by radiologists [18], and the interpretation of radiological imaging [19] in addition to the impact of including age and gender in DR screening algorithms [20].

AI algorithms have been touted as a means of improving health care access in low-resource settings [21]. Many existing algorithms have been developed from images obtained from only the United States, Europe, and China. There is a near lack of such data from the African continent raising concerns about generalizability, accuracy, and bias [22]. However, collecting even basic clinical information in low-resource settings is difficult, as existing medical records typically have less detailed information than those in high-resource settings and may be paper-based; the available results and findings are often incomplete and less accurate. Prospective clinical metadata collection at the time of image capture is also limited by patient health literacy and knowledge about their health conditions.

### Project Objective

Despite the importance of high-quality labels for optimizing algorithm performance [23], there is little guidance in the literature about how to collect and use clinical metadata for image labeling in low-resource settings. The Multimodal Database of Retinal Images in Africa (MoDRIA) is one of the inaugural research projects of the Mbarara University Data Science Research Hub (MUDSReH) [24], part of the Data Science for Health Discovery and Innovation in Africa (DS-I Africa) [25] initiative to “advance Data Science and related innovations in Africa to create an ecosystem that can begin to provide local solutions to countries’ most immediate public health problems through advances in research.” As a critical step in the development of the MoDRIA database, we aim to understand how the presence or absence of clinical metadata influences how labelers annotate retinal images. These images are used to develop AI algorithms so it is important to determine if the labeling process introduces a source of bias that may impact the accuracy of algorithms. Here, we present an analysis to determine whether the availability of clinical metadata during the image labeling process influences the accuracy, sensitivity, and specificity of image labels provided by newly trained labelers when using a known set of properly labeled images.

## Methods

### Setting

This project was conducted at the Mbarara University of Science and Technology (MUST) in Mbarara, Uganda in November 2023. MUST is the site of the MUDSReH and the MoDRIA research project. MUST is also the parent institution for the Mbarara Regional Referral Hospital in southwestern Uganda and is located 268 kilometers southwest from the capital of Kampala.

### Project Participants and Recruitment

The project participants were 20 Ugandan preinterns recruited from MUST medical school graduates awaiting the commencement of their internship. Participation was voluntary. Inclusion criteria included completion of an imaging labeling training workshop and willingness to participate and follow study procedures, these “MoDRIA labelers” completed a labeling training course consisting of 40 hours of teaching, training, supervised labeling, and testing by Ugandan ophthalmologists and ophthalmology residents, and 2 international visiting retinal specialists. The training course content included (1) a review of the Brazilian Diabetic Retinopathy fundus image dataset (BRSET) image reading training manual [26] and (2) videos and didactic lectures on retinal anatomy, ME, DR abnormalities in each ICDR category, and ME and a 4-day hands-on workshop in which MoDRIA labelers practiced labeling a minimum of 200 CFPs followed by tests to confirm labeler competency and accuracy by test set labeling. The labeling activities took place in a conference room. Each participant used a separate laptop and could take as much time as necessary to label each image.

### Data Collection

#### Metadata

This project used clinical metadata only and included blood pressure, visual acuity, blood glucose, the presence of diabetes, hypertension, or HIV, and the class of medications taken. To ensure all metadata elements were available for all test images, metadata values were synthesized to align with the ICDR scores of the test image. The metadata for each image was presented in a spreadsheet with the image number and fields to enter the ICDR score and ME assessment. The images appeared on the same screen.

#### Image Sets

The MoDRIA database contains 14,000 CFPs from 3500 individuals. Each study participant has 4 CFPs (disc center and macular center view from right and left eyes). For quality assessment, we established that an image was adequate when the area of interest fell within predefined limits, and the visible image was of sufficient quality for grading purposes. Specifically, we ensured that the fovea center is positioned greater than 2 disc diameters away from the image edge [27]. The MoDRIA database will be used to develop AI algorithms to screen patients for posterior segment retinal diseases such as DR. The MoDRIA CFP labeling protocol was based on the BRSET labeling protocol [28]. It is a publicly available collection of 16,000 retinal fundus images collected and labeled in Brazil.

MoDRIA CFPs were collected on 3-Nethra Classic (Forus Royal) fundus cameras by ophthalmic technicians trained in fundus photography. BRSET images were collected on a Nikon NF505 (Nikon) and a Canon CR-2 (Canon Inc) in JPEG format, and no preprocessing techniques were applied. There were 50 CFPs in the test set for this study, 20 from MoDRIA and 30 from the BRSET. The ICDR and ME scores of the BRSET and MoDRIA test set images were reviewed and confirmed by the international retina specialists participating in the study (LN and MM). The distribution of ICDR scores and the presence or absence of DME in the test set is presented in Table 2, with approximately half the images being normal.

**Table 2:** Referability and nonreferability of color fundus photos used in labeling test set based on International Classification of Diabetic Retinopathy (ICDR) scores and macular edema (N=50, each image scored for ICDR and macular edema).

Scores				Images, n	Macular edema		
**Nonreferable (n=28)**							
	**ICDR ≤1**						
		0	26			Absent	
		1	4			40	
**Referable (n=22)**							
	**ICDR >2**						
		2	6				Present
		3	6				10^a^
		4	8				—^b^

^a^Two images had ICDR ≤1 with the presence of macular edema so included in the referrable category.

^b^Not applicable.

### Research Design

#### Labeling Protocol

Each CFP was individually labeled for DR with an ICDR score of 0-4, ranging from 0 (no retinopathy) to 4 (proliferative DR; Table 1). These scores were grouped into 2 categories, that are (1) nonreferable (ICDR ≤1 and no ME) and (2) referrable (ICDR >2 and with ME). The same CFPs were also labeled with the presence or absence of ME.

#### Image Labeling by Preinterns

**Figure 1:**
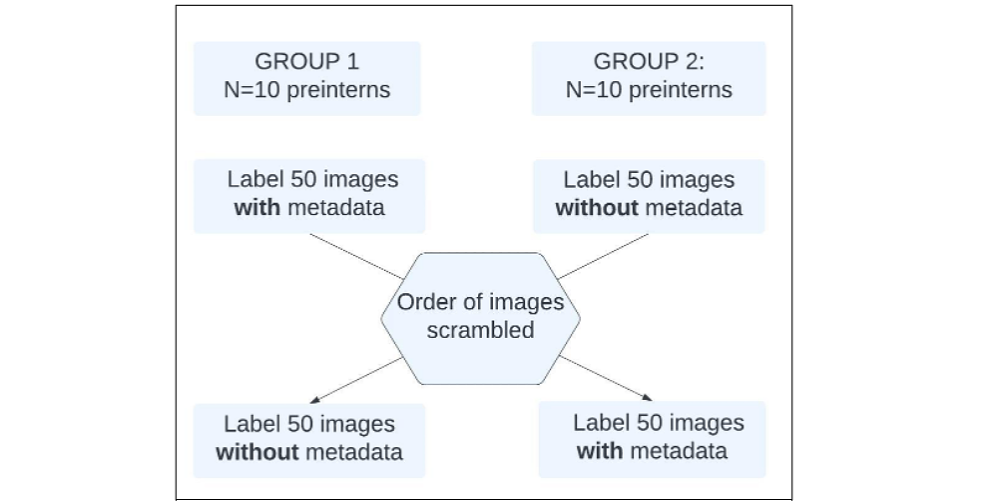
Crossover study design diagram for retinal image labeling groups with and without metadata.

This is a crossover assessment with 2 groups and 2 phases. Each group had 10 randomly assigned preintern labelers who labeled the same image test set of 50 CFP twice (Phase 1 and Phase 2) with the ICDR score and presence or absence of ME. Group 1 (“with or without”) labeled the CFPs with metadata in Phase 1 and without metadata in Phase 2. Group 2 (“without or with”) labeled the CFP in Phase 1 without metadata and with metadata in Phase 2. In Phase 2, the order of presentation for the same CFPs was scrambled for both groups (Figure 1). After labeling the test set images with and without metadata, the results of ICDR scores and the presence or absence of ME were recorded for each participant. The sensitivity and specificity of referrable and nonreferrable DR with and without access to clinical metadata were calculated, using the test image labels as the gold standard.

#### Statistical Analysis

Statistical analysis was conducted using STATA (version 17.0; StataCorp LLC). ICDR scores were grouped into referable (ICDR 2-4 with or without ME) and nonreferable categories (ICDR 0-1 and no ME) for statistical analysis. Specificity and sensitivity of referable retinopathy were based on ICDR scores and ME calculated using a 2-sided *t* test. Comparison of sensitivity and specificity for ICDR and ME with and without metadata for each participant was calculated using the signed rank test. Statistical significance was set at *P*<.05.

### Ethical Considerations

This work was part of the ongoing MoDRIA study (MUST IRB approval number: MUST-2021-239 and Uganda National Council of Science and Technology number: HS2094ES) as a quality improvement project to improve the training of CFP readers and optimize the labeling protocol of the MoDRIA fundus image database Uganda.

## Results

### Overview

Table 3 lists the sensitivity and specificity of referable retinopathy based on both ICDR scores calculated with and without metadata. The sensitivity and specificity of the ICDR score and the presence or absence of ME were also calculated for the 20 individual labelers with and without metadata (Multimedia Appendix 1). There were no statistically significant differences with and without metadata for any of the labelers.

**Table 3:** Comparison of sensitivity and specificity of labeling color fundus photo as referable or nonreferable with and without metadata for all labelers (N=20).

Diagnostic measure		With metadata	No metadata	*P* value		
ICDR^a^: referable versus nonreferable, mean (95% CI)						
	Sensitivity	92.8 (87.6-98.0)	93.3 (87.6-98.9)	.90		
	Specificity	84.9 (75.1-94.6)	88.2 (79.5-96.8)	.84		
Macular edema: present versus absent, mean (95% CI)						
	Sensitivity	64.3 (57.6-71.0)	63.1 (53.4-73.0)	.60		
	Specificity	86.5 (81.4-91.5)	87.7 (83.9-91.5)	.69		

^a^ICDR: International Classification of Diabetic Retinopathy.

### Diabetic Retinopathy

The sensitivity for identifying referrable DR with metadata was 92.8% (95% CI 87.6-98.0) compared with 93.3% (95% CI 87.6-98.9) without metadata, and the specificity was 84.9% (95% CI 75.1-94.6) with metadata compared with 88.2% (95% CI 79.5-96.8) without metadata. The improvements in sensitivity and specificity without metadata were not statistically significant.

### Macular Edema

The sensitivity for identifying the presence of ME was 64.3% (95% CI 57.6-71.0) with metadata, compared with 63.1% (95% CI 53.4-73.0) without metadata, and the specificity was 86.5% (95% CI 81.4-91.5) with metadata compared with 87.7% (95% CI 83.9-91.5) without metadata. The improvements in sensitivity and specificity without metadata were not statistically significant.

## Discussion

### Principal Results

The objective of our project was to determine if access to clinical metadata influences how labelers annotate for DR and ME. This information can help understand potential sources of bias in the labeling process. This assessment serves as a baseline for future iterative improvements in the training of labelers and the labeling process. Our results can also inform a more rigorous investigation of the role of metadata in the labeling process for the MoDRIA data set as well as other data sets developed through MUDSReH, the DS-I for Africa, and others.

As a group, the labelers detected referrable DR reasonably well (92.8%) but detected ME only 64.3% of the time. This difference may be a result of the subtle appearance of hard exudates on ME when there are only cystic changes or a blunted foveal reflex rather than the presence of more obvious lipids. In screening programs, the false negative rate (failing to identify the condition when it is present) is the most potentially dangerous error. Given the more subtle presentation on ME CFPs, optical coherence tomography, which easily identifies ME, is a valuable complementary tool to CFPs in screening for referrable DR if available.

Overall, the sensitivity and specificity scores tended to be slightly better without metadata, but the difference was not statistically significant. The wide confidence intervals noted in the data reflect the variation in our labelers. We cannot make definitive conclusions about whether knowing the clinical metadata ahead of determining the labels may have introduced bias on the part of the labelers, which could impact the sensitivity and specificity of image labels in our study. Another consideration is whether knowing the metadata ahead of determining the labels may have introduced bias on the part of the labelers. For example, if the labeler sees the individual has a history of diabetes and elevated blood glucose, they may be more likely to give a higher ICDR score. However, given the importance of metadata in clinical situations we believe that it may benefit labeling quality as well. For example, mild DR, hypertensive retinopathy, and HIV retinopathy can have a similar appearance on CFP and be difficult to differentiate with just a single image.

Understanding how clinical metadata influences the annotation decisions of image labelers is important as supervised machine learning algorithms for labeling are evolving and clinical metadata has been shown to influence outcomes [29,30]. Another key consideration is the development of an algorithm development using multimodal data, for example, images, and clinical and demographic information. The evolution of AI algorithms will inevitably incorporate the fusion of such multimodal data streams, harnessing the capabilities of natural language processing, computer vision, and tabular data analysis, akin to the intricate layers of clinical decision-making.

### Comparison With Previous Work

Few other studies have been published on the impact of using metadata in labeling CFP. We conducted a MEDLINE search using Medical Subject Headings: “fundus image” and “metadata,” “Image grading” and “metadata,” “fundus photo” and “metadata,” and “image grading” and “clinical information” to search for previous studies evaluating the impact of using metadata or clinical information in the CFP labeling process. Additional free text topic heading searches with the same terms were also conducted without finding other dedicated studies using metadata in the CFP labeling process. We also examined the labeling protocols for the following large open-source fundus photo data sets- Messidor [31], BRSET [28], Eye Pacs [32], and IDRiD [33] and did not find documentation indicating whether metadata was used or not used in the labeling process.

### Limitations

We acknowledge several important limitations of our project. First, our assessment design did not include a defined step in the process where the labelers confirmed a review of the metadata. It was provided on the screen at the time of labeling, and they were encouraged to use it, but there was no step confirming whether it was viewed. Second, we selected a sample size of 50 images, which may not have been large enough given that half the images were normal examinations. This distribution of ICDR categories was intentionally chosen to better reflect the composition of the MoDRIA database; however, it may have introduced some bias as the distribution across categories was not even. Third, the focus of labeler training was to familiarize themselves with CFPs of normal and DR images, as well as other common retinal pathology. The use of metadata to inform labeling decisions tended to be subsumed by learning retinal image pathology. This process may have influenced if and how they used the metadata. Fourth, the images were labeled with ICDR scores 0-4, but our analysis was based on a binary classification of referable or nonreferable DR. Finally, our metadata was synthesized based on the ICDR score and the presence or absence of ME therefore may not be the same as using available clinical metadata.

### Strengths

Our project also has several strengths. To our knowledge, it is the first attempt to understand the role of metadata in CFP image labeling by a cadre of nonophthalmologists in Africa. It is critically important to build local image labeling capacity to support the development and implementation of data science research and technologies in Africa and avoid the expansion of digital sweatshops in Africa [34]. It also provided experience using a quality improvement approach to improve image labeling and training for the researchers and clinicians at the MUDSReH. An advantage of a quality improvement approach is the ability to rapidly identify actionable results, such as the need for additional training on recognizing ME. Finally, this project highlighted the importance of understanding metadata and the need to conduct further rigorous investigations.

### Opportunities for Improvement and Future Study

As this was a quality improvement project, we sought opportunities for improvement in our labeling process. Specifically, we identified the items, that consist of (1) defined guidelines for reviewing metadata in the labeling process, including when it should be reviewed; (2) adding a field confirmation review of metadata in the MoDRIA data collection and management application developed by the MUDSReH hub team; and (3) enhanced training on appearance of ME on CFP. We also identified several areas for future study. First, we intend to perform a more rigorous, sufficiently powered study to determine the sensitivity and specificity of CFP labels with and without metadata using a cohort of images from patients with DM without HIV or hypertension with a higher percentage of abnormal images. This approach will also allow analysis by individual ICDR scores rather than referable or nonreferable categories, so we have a more nuanced understanding of the impact of metadata on labels and algorithm performance. Given the challenge of metadata collection in this low-resource environment, we also plan to determine which metadata variables are most informative in accurately predicting referrable DR. Finally, we will assess the optimal timing and method to present metadata to labelers, as well as determine intrarater reliability with and without metadata.

### Conclusion

In this quality improvement project, clinical metadata availability did not influence labeling quality. Additional studies are needed to understand the potential implications of the process and components of labeling with and those without metadata more thoroughly with regard to accuracy and bias. These issues have far-reaching implications given the rapidly expanding use of AI with clinical images, including on the African continent.

## Appendix

Multimedia Appendix 1 Individual reader sensitivity and specificity results.

## Abbreviations

AI: artificial intelligence
BRSET: Brazilian Diabetic Retinopathy fundus image dataset
CFP: color fundus photos
DR: diabetic retinopathy
DS-I Africa: Data Science for Health Discovery and Innovation in Africa
ETDRS: Early Treatment Diabetic Retinopathy Study
ICDR: International Classification of Diabetic Retinopathy
ME: macular edema
MoDRIA: Multimodal Database of Retinal Images in Africa
MUDSReH: Mbarara University Data Science Research Hub
MUST: Mbarara University of Science and Technology

## Data Availability

The data sets generated during and/or analyzed during this study are available from the corresponding author on reasonable request.
