# Supplementary material for: Assessment of Clinical Metadata on the Accuracy of Retinal Fundus Image Labels in Diabetic Retinopathy in Uganda: Case-Crossover Study Using the Multimodal Database of Retinal Images in Africa
Source: JMIR Form Res. 2024 Sep 18;8:e59914. doi: 10.2196/59914 (PMC11451581; doi:10.2196/59914)
Supplement: Multimedia Appendix 1 [file formative_v8i1e59914_app1.docx]

**Multimedia appendix 1: Individual reader sensitivity and specificity results**

| Labeler ID | ICDR Score | | | | | | Presence or Absence of Macular Edema | | | | | |
| --- | --- | --- | --- | --- | --- | --- | --- | --- | --- | --- | --- | --- |
|  | Sensitivity | | P-value | Specificity | | P-value | Sensitivity | | P-value | Specificity | | P-value |
|  | With metadata | Without  metadata |  | With metadata | Without  metadata |  | With metadata | Without  metadata |  | With metadata | Without  metadata |  |
| 1 | 95 | 100 | 0.32 | 73.3 | 90 | 0.32 | 50 | 18.2 | 0.32 | 84.6 | 97.4 | 0.32 |
| 2 | 90 | 100 | 0.32 | 100 | 90 | 0.32 | 54.6 | 45.5 | 0.32 | 94.9 | 87.2 | 0.32 |
| 3 | 100 | 100 | - | 100 | 100 | - | 63.6 | 72.7 | 0.32 | 97.4 | 100 | 0.32 |
| 4 | 100 | 100 | - | 93.3 | 93.3 | - | 90.0 | 72.7 | 0.32 | 71.8 | 74.4 | 0.32 |
| 5 | 89.5 | 100 | 0.32 | 73.3 | 96.7 | 0.32 | 72.7 | 90.9 | 0.32 | 76.9 | 82.1 | 0.32 |
| 6 | 100 | 100 | - | 93.3 | 90 | 0.32 | 90.9 | 63.6 | 0.32 | 76.9 | 79.5 | 0.32 |
| 7 | 100 | 100 | - | 76.7 | 73.3 | 0.32 | 72.7 | 90.9 | 0.32 | 79.5 | 79.5 | - |
| 8 | 100 | 100 | - | 80 | 80 | - | 81.8 | 63.6 | 0.32 | 89.7 | 92.3 | 0.32 |
| 9 | 96.7 | 100 | 0.32 | 98 | 96.7 | 0.32 | 45.5 | 45.5 | 0.32 | 100 | 97.4 | 0.32 |
| 10 | 100 | 100 | - | 10 | 20 | 0.32 | 72.7 | 90.9 | 0.32 | 55.3 | 74.4 | 0.32 |
| 11 | 100 | 100 | - | 93.3 | 96.7 | 0.32 | 63.6 | 63.6 | 0.32 | 92.3 | 89.7 | 0.32 |
| 12 | 90 | 90 | - | 89.7 | 93.3 | 0.32 | 72.7 | 45.5 | 0.32 | 87.2 | 82.1 | 0.32 |
| 13 | 75 | 75 | - | 100 | 100 | - | 54.6 | 72.7 | 0.32 | 92.3 | 87.2 | 0.32 |
| 14 | 85 | 95 | 0.32 | 66.7 | 93.3 | 0.32 | 72.7 | 81.8 | 0.32 | 79.5 | 89.7 | 0.32 |
| 15 | 100 | 90 | 0.32 | 96.7 | 96.7 | - | 54.6 | 72.7 | 0.32 | 87.2 | 92.3 | 0.32 |
| 16 | 100 | 100 | - | 96.7 | 100 | 0.32 | 72.7 | 72.7 | - | 92.3 | 94.9 | 0.32 |
| 17 | 90 | 90 | - | 83.3 | 86.7 | 0.32 | 54.6 | 72.7 | 0.32 | 89.7 | 94.9 | 0.32 |
| 18 | 95 | 95 | - | 100 | 100 | - | 45.5 | 18.2 | 0.32 | 100 | 84.6 | 0.32 |
| 19 | 55 | 50 | 0.32 | 73.3 | 66.7 | 0.32 | 45.5 | 54.6 | 0.32 | 87.2 | 76.9 | 0.32 |
| 20 | 95 | 95 | - | 100 | 100 | - | 54.6 | 54.6 | - | 94.9 | 97.4 | 0.317 |
